# Thermooxidation of Four Sartans: Kinetic Analysis Based on Thermo-Gravimetric Data

**DOI:** 10.3390/molecules29235527

**Published:** 2024-11-22

**Authors:** Adriana Ledeţi, Bianca Baul, Amalia Ridichie, Denisa Ivan, Titus Vlase, Carmen Tomoroga, Anca Dragomirescu, Gabriela Vlase, Răzvan Adrian Bertici, Dana Emilia Man, Ionuţ Ledeţi

**Affiliations:** 1 Advanced Instrumental Screening Center, Faculty of Pharmacy, Victor Babeş University of Medicine and Pharmacy, 2 Eftimie Murgu Square, 300041 Timisoara, Romania; afulias@umft.ro (A.L.); amalia.ridichie@umft.ro (A.R.); circioban.denisa@umft.ro (D.I.); dragomirescu.anca@umft.ro (A.D.); ionut.ledeti@umft.ro (I.L.); 2 Faculty of Industrial Chemistry and Environmental Engineering, University Politehnica Timisoara, 2 Victoriei Square, 300006 Timisoara, Romania; bianca.baul@student.upt.ro; 3 Research Centre for Thermal Analysis in Environmental Problems, West University of Timisoara, Pestalozzi Street 16, 300115 Timisoara, Romania; titus.vlase@e-uvt.ro (T.V.); gabriela.vlase@e-uvt.ro (G.V.); 4 Faculty of Medicine, Victor Babeş University of Medicine and Pharmacy, 2 Eftimie Murgu Square, 300041 Timisoara, Romania; razvan.bertici@umft.ro (R.A.B.); man.dana@umft.ro (D.E.M.)

**Keywords:** angiotensin II receptor antagonists, sartan, kinetic analysis, thermal stability

## Abstract

Angiotensin II receptor antagonists are tetrazole derivatives used in the treatment of high blood pressure, and are also indicated for the treatment of heart failure (NYHA class II-IV). They are used alone or in combination with other classes of antihypertensives or diuretics for the effective management of high blood pressure. In this study, we aim to evaluate the thermal stability and degradation kinetics for the principal compounds used in therapy from this class, namely telmisartan, valsartan, olmesartan medoxomil, and losartan potassium. To obtain the thermoanalytical data for the kinetic investigations, the TG and DTG curves were registered at five different heating rates (β = 2, 4, 6, 8, and 10 °C min^−1^). The kinetic methods used were a preliminary ASTM E698 method and two isoconversional methods: Flynn–Wall–Ozawa and Friedman. For each molecule, the results showed complex decomposition processes consisting of complex reaction sequences.

## 1. Introduction

Angiotensin II receptor antagonists (sartans) are tetrazole derivatives used in the treatment of high blood pressure, and are also indicated for the treatment of heart failure (NYHA class II-IV) in adults with left ventricular systolic dysfunction (ejection fraction = 40%) to reduce hospitalizations and death from cardiovascular causes [1,2,3]. They are used alone or in combination with other classes of antihypertensives or diuretics, e.g., hydrochlorothiazide, for the effective management of high blood pressure [1,2,3,4].

As their mechanism of action, they bind selectively to the angiotensin type 1 receptor (AT1), preventing the binding of angiotensin II protein (a protein which exerts a hypertensive effect, by vasoconstriction, stimulation and synthesis of aldosterone and antidiuretic hormone, cardiac stimulation, and the increase of renal sodium reabsorption). Thus, sartans present a protective effect on the heart by improving the cardiac function, reducing the afterload and increasing the cardiac output, and preventing ventricular hypertrophy and remodeling. This class of compounds also affects the renin–angiotensin–aldosterone system, which plays an important role in hemostasis and regulation of renal, vascular, and cardiac functions [5,6].

After oral administration, the active substances are quickly absorbed, but their bioavailability is low, due to the effect of the first hepatic passage. To increase their bioavailability (from 4.5% to 28.6% in the case of olmesartan), esterified forms of the drugs have been developed; for example, olmesartan is available as a prodrug in the form of olmesartan medoxomil, which is rapidly transformed in vivo in pharmacologically active olmesartan; also, losartan is conditioned as a potassium salt, called losartan potassium. Binding to plasma proteins is strong (>99%) and constant at plasma concentrations well above the range achieved with recommended doses. Regarding the half-life, there are differences between the molecules from this class: for olmesartan it is 13 h, for telmisartan it is 24 h, for losartan between 1.5 and 2.5 h (it presents a more potent metabolite, namely the E3174 metabolite, for which the half-life is between 6 and 9 h), and for valsartan between 6 and 9 h. Elimination is mainly in the unchanged form, predominantly biliary and digestive, with a small part of the administered dose being excreted in the urine [5,7,8,9,10,11]. The physicochemical properties of the selected sartans are presented in Table 1.

**Figure 1:**
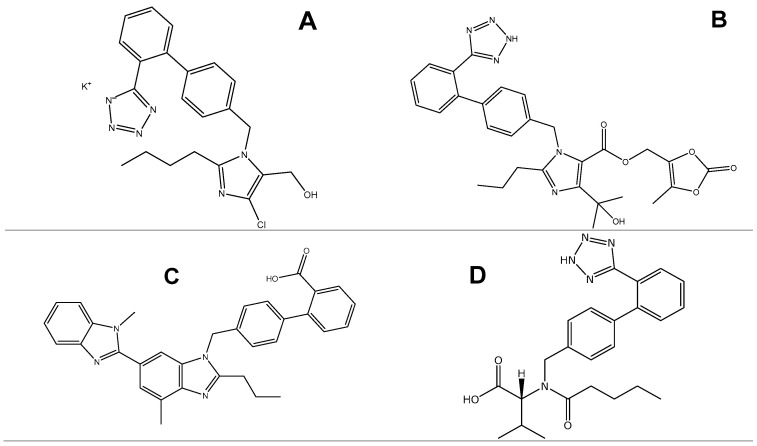
Chemical structures of the selected sartans: (**A**) LOS, (**B**) OLM, (**C**) TELM, (**D**) VLS.

The aim of this study was to evaluate the thermal stability and degradation kinetics for a series of sartans, namely telmisartan (TELM), valsartan (VLS), olmesartan medoxomil (OLM), and losartan potassium (LOS), to gather information regarding the thermal stability of the principal compounds used in therapy from this class. The kinetic methods used were a preliminary ASTM E698 method and, respectively, two isoconversional methods: Flynn–Wall–Ozawa (FWO) and Friedman (FR). Also, to obtain the kinetic triplet and for a better understanding of the mechanism underlying the degradation process, the modified non-parametric (NPK) kinetic method was applied. The chemical structures of these sartans are shown in Figure 1. Considering that TELM does not present a tetrazole ring, it presents some differences in terms of pharmacokinetic properties, namely having the highest affinity for the AT1 receptor among the available angiotensin II receptor blockers and the lowest affinity for the angiotensin type 2 receptor (AT2). TELM may also have PPAR γ (peroxisome proliferator-activated receptor gamma) agonist properties that could confer beneficial metabolic effects, as PPAR γ is a nuclear receptor that regulates specific gene transcription and whose target genes are involved in the regulation of glucose and lipid metabolism, as well as in anti-inflammatory responses [1,4,17].

## 2. Results and Discussion

### 2.1. Results of the Thermal Investigations

**Figure 2:**
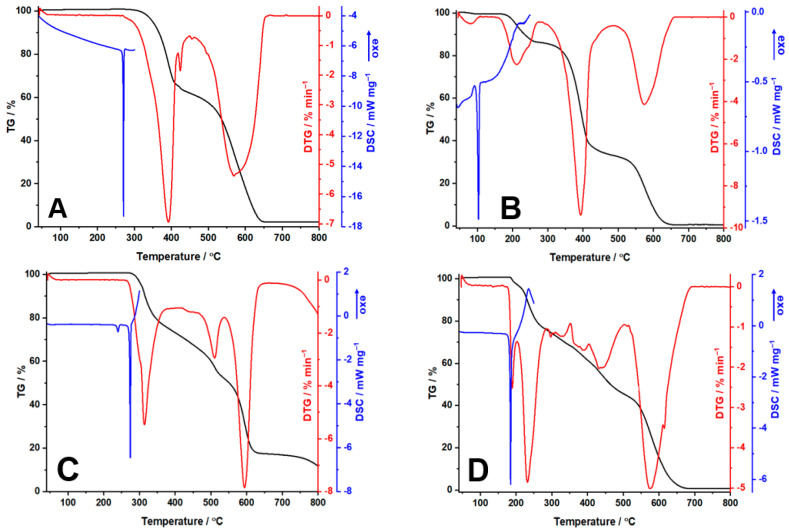
Thermoanalytical curves of (**A**) TELM, (**B**) VLS, (**C**) LOS, (**D**) OLM.

In Figure 2, the obtained thermoanalytical curves (TG/DTG and DSC) are represented, while in Table 2 the interpretation of the curves can be observed.

TELM (Figure 2A) shows high thermal stability (up to 274 °C) when the melting process highlighted by the DSC curve between 264 and 277 °C with a peak at 271 °C (an endothermic event) accompanies the beginning of the mass loss, so there is a phase transition accompanied by decomposition. The decomposition process takes place in two steps identifiable on the TG/DTG curves. The first process takes place in the range of 274–419 °C with a mass loss of ~40%. The second decomposition process commences at 450 °C and ends at 668 °C, presenting a Δm = 59.7%.

For VLS on the TG/DTG curves, four steps of the decomposition process are observed (Figure 2B). The first process, which starts at an ambient temperature, represents the loss of the absorbed water (Δm = 0.5%). The endothermic peak observed at 103 °C indicates the melting temperature of VLS, in good agreement with the data presented in two patents [18,19]. The first degradation process of VLS starts at 148 °C and continues until 277 °C, presenting a maximum on the DTG curve at 212 °C and a slight loss of its mass. The second process starts at 284 °C, and is the major decomposition step considering that VLS loses more than half of its mass. The third step of decomposition of anhydrous VLS is between 486 and 662 °C, highlighted on the DTG curve by one peak at 575 °C.

Regarding LOS, thermal stability up to 258 °C can be observed, as well as a degradation process consisting of three steps (Figure 2C). The first one is characterized by Δ = 25.4%, accompanied on the DSC curve by two endothermic peaks at 240 and 275 °C. The first endothermic event can be associated with an enantiotropic polymorphic transition, as presented in the literature [20], while the second one represents the melting point of losartan potassium, in good agreement with the data presented in the literature. The second degradation step begins at 418 °C, presenting a loss of 19.3% of its mass and one maxim on the DTG curve at 510 °C. The third degradation step is between 538 and 652 °C, correlated with a loss of ~35% of the sample mass.

In the case of OLM decomposition (Figure 2D), five distinct processes could be identified in the selected temperature range. The first one, which takes place in the range 171–202 °C, corresponds to a dehydration process (theoretical water content 0.5 mol/mol OLM, determined water content 0.52 mol/mol OLM), being a result in agreement with the hemihydrate mentioned in patent EP1801111 [21]. It is known that the dehydration of pharmaceutical active hydrates generally occurs at temperatures close to the normal boiling temperature of water, considering that the hydrogen bonds that are formed in the molecular network are usually broken at this temperature [22]. For this hemihydrate, it is observed that the loss of water from the network occurs at considerably higher temperatures, indicating the strong binding of it in the crystalline network. In the same temperature range, an endothermic event can be observed on the DSC curve, which characterizes the melting point of OLM (peak at 185 °C). The following processes observed represent the decomposition of OLM. It can be concluded that OLM is thermally stable up to 202 °C, the temperature at which it begins to degrade, losing up to ~22% of its mass. Afterwards, the degradation continues with a slight loss of mass (7.4%) between 287 and 353 °C. Later, two major decompositions are observed in the temperature ranges of 353–504 °C and 504–690 °C, with losses of mass of 22.9% and ~45%, respectively.

### 2.2. Results of the Kinetic Investigations

For the kinetic investigations for each sartan, the first degradation process noticed on the DTG curve was selected, with an exception for OLM, for which two degradation processes were investigated, the dehydration and the decomposition of OLM. In Table 3 are presented the selected temperature ranges for each sartan.

ASTM E698 is a non-isoconversional kinetic method based on the Arrhenius equation, being described as a linear regression correlating the conversion degree to the heating rate [23]. It is used only as a preliminary method, since it is more suitable for single-step processes [24,25,26,27]. The following is the equation used to determine the activation energy:

(1) $$\beta \frac{d\alpha }{\mathrm{dT}}=k_{o}\exp\left(\frac{E_{a}}{\mathrm{RT}}\right)\left(1-\alpha \right)$$

FWO is an isoconversional integral kinetic model which permits the calculation of the activation energy without knowing the reaction order. In order to obtain the value of it the Doyle approximation is used, the equation being as follows [28,29,30,31,32,33]:

(2) $$\log\beta =\log\frac{{\mathrm{AE}}_{a}}{g\left(\alpha \right)R}-2.315-0.457\frac{E_{a}}{\mathrm{RT}}$$

By plotting the FWO method (the natural logarithm from the heating rate vs. 1/T), parallel lines are obtained for each conversion degree (from 0.01 to 0.99), and the activation energy is determined from the slope of each line [28].

The FR kinetic model is an isoconversional differential method considered to be the most accurate one, since no approximation is used to calculate the value of the activation energy. It is determined after the plotting of ($\ln\frac{d\alpha }{\mathrm{dt}}$ vs. 1/T) and with the help of the following equation [25,26,34,35,36]:

(3) $$\ln{\left(\frac{d\alpha }{\mathrm{dt}}\right)}_{\alpha ,t}=\ln\left[A_{\alpha }f\left(\alpha \right)\right]-\frac{E_{a}}{{\mathrm{RT}}_{\alpha ,t}}$$

The NPK method is a kinetic model that does not use any approximation and in which the reaction rate results from two independent functions: f(α), a function of the degree of conversion, and k(T), a temperature dependence. Furthermore, this method indicates the type of processes that take place during the degradation (chemical or physical), with the help of the reaction orders (m and n), as indicated by the model of Šesták and Berggren:

(4) $$f\left(\alpha \right)=\alpha^{m}\;{\left(1-\alpha \right)}^{n}$$

Another advantage of this kinetic model is that it provides the value of the variance, which gives information regarding the contribution of each process to the global degradation process [37,38,39].

In Table 4 are represented the values of the activation energy obtained for each sartan by employing the FWO and FR isoconversional kinetic methods.

**Figure 3:**
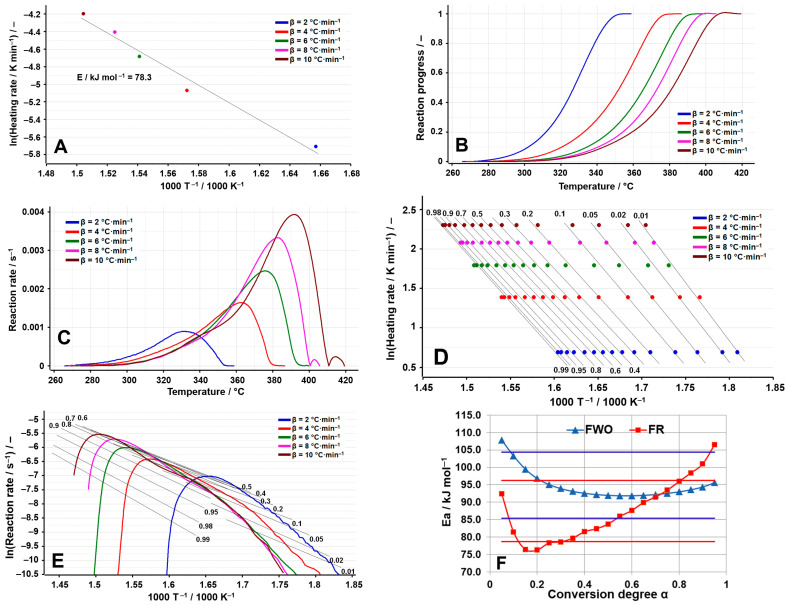
Results of the kinetic analysis performed on TELM: (**A**) kinetic method ASTM E698; (**B**) reaction progress vs. temperature; (**C**) reaction rate vs. temperature; (**D**) kinetic method FWO; (**E**) kinetic method Fr; (**F**) E_a_ vs. α according to the FWO and FR methods.

The thermal decomposition process that was investigated in the case of TELM (Figure 3A–F) is the one that takes place between 266 and 359 °C at β = 2 °C min^−1^, a process that moves to a higher temperature with the increase in the heating rate, reaching the range of 274–419 °C at β = 10 °C min^−1^. The ASTM E698 method indicates an activation energy of 78.3 kJ mol^−1^, which highlights the relatively low stability of TELM in relation to the other sartans analyzed, as can be seen from Table 4.

Thus, for TELM, the results of the isoconversional methods disagree with those of the preliminary method (ASTM E698), indicating considerably higher activation energies. From the analysis of the variation in E_a_ vs. α, it is observed that most of the individual values of E_a_ are found within the deviation limit of ±10% around the mean; thus, the FWO method presents at the beginning of the process higher activation energies, which then tend towards a smaller value, while the FR method indicates the minimum of the activation energy at α = 0.2, after which a sharp increasing tendency towards the end of the investigated process can be noted, with the values obtained for α > 0.80 being outside the selected range. This tendency suggests a change in the decomposition mechanism with the Increase in the heating rate, observations also supported by the aspect of the dependence of the reaction rate on temperature (Figure 3C), which indicates at temperatures above 400 °C (for the processes recorded at β = 8 °C min^−1^ and β = 10 °C min^−1^) the appearance of a secondary process of a much lower amplitude than the primary process. The complexity of the decomposition process is also suggested by the value of the difference between E_max_ and E_min_, which is greater than 20% of the average value for the activation energy only for the Friedman method, since this method is a differential one and analyzes the process point by point, unlike the FWO method, which is an integral isoconversional method that provides an overview of the entire process analyzed (see Table 5).

For TELM, it can be observed that two simultaneous processes take place during the degradation, the value of the activation energy being the result of the following equation:

(5) $$\bar{E}=\lambda_{1}\cdot E_{1}+\lambda_{2}\cdot E_{2}$$

**Figure 4:**
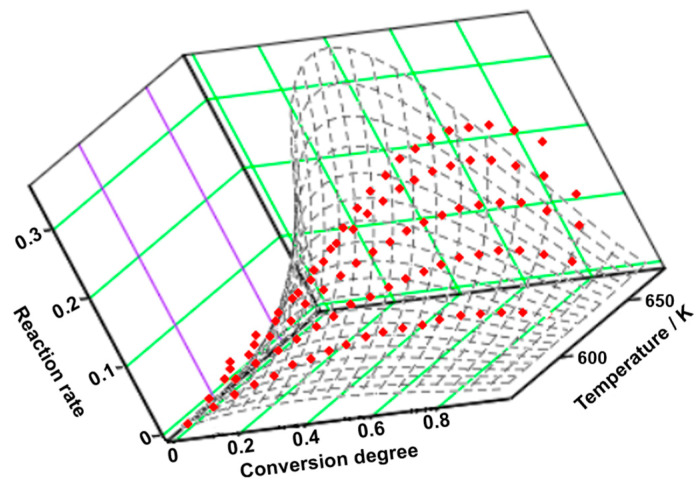
The 3D transformation surface for TELM from the NPK method.

The experimental points obtained at five heating rates are represented in a tridimensional system and interpolated as a continuous reaction rate surface (see Figure 4). The first process contributes the most to the degradation process (λ = 87.5%), consisting of both physical (m = 1) and chemical (n = 1/2) transformations. Considering that the value of the variance of the second process is significantly lower than that of the first process, its contribution to the global process is minor. During this step, both physical (m = 3/2) and chemical (n = 1) transformations are noted (see Table 6).

**Figure 5:**
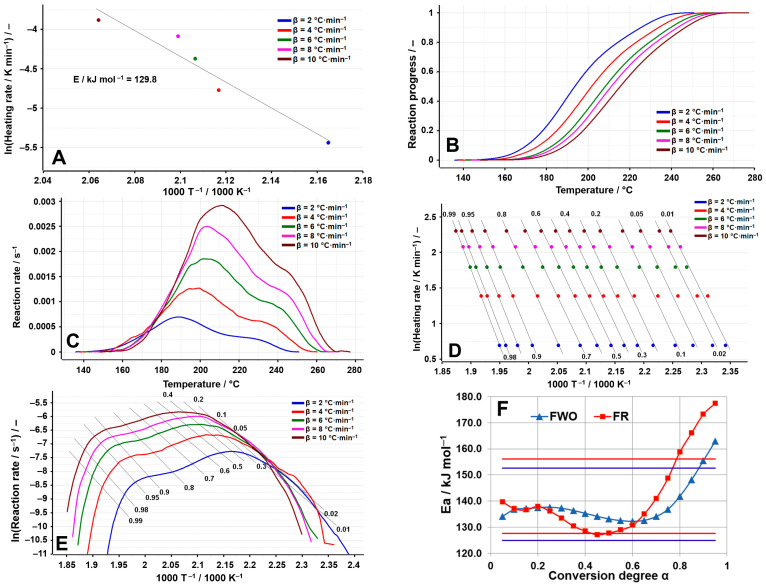
Results of the kinetic analysis performed on VLS: (**A**) kinetic method ASTM E698; (**B**) reaction progress vs. temperature; (**C**) reaction rate vs. temperature; (**D**) kinetic method FWO; (**E**) kinetic method Fr; (**F**) E_a_ vs. α according to the FWO and FR methods.

In the case of VLS (Figure 5A–F), the ASTM E698 method presents a greater dispersion of the experimental points, which is an indication of the change in the decomposition mechanism with the increase in the heating rate. This is also supported by the other data obtained: the sigmoid that represents the variation in the reaction progress vs. temperature (Figure 5B) is irregular, similar to the dependence of the reaction rate on temperature, which presents a secondary process characterized by a local maximum between 220 and 270 °C. The FWO and FR methods confirm the existence of a complex decomposition process, consisting of several overlapping processes, the concave aspect of E_a_ vs. α indicating a process with a reversible stage (it decreases with α until α = 0.45 (FR) or until α = 0.60 (FWO)), followed by a thermooxidation process involving parallel or consecutive reactions (it increases strongly with α until the end of the investigated process) [41]. This aspect is also represented by the values of the difference between E_max_ and E_min_, which are greater than 20% of the average value for the activation energy (Table 5).

**Figure 6:**
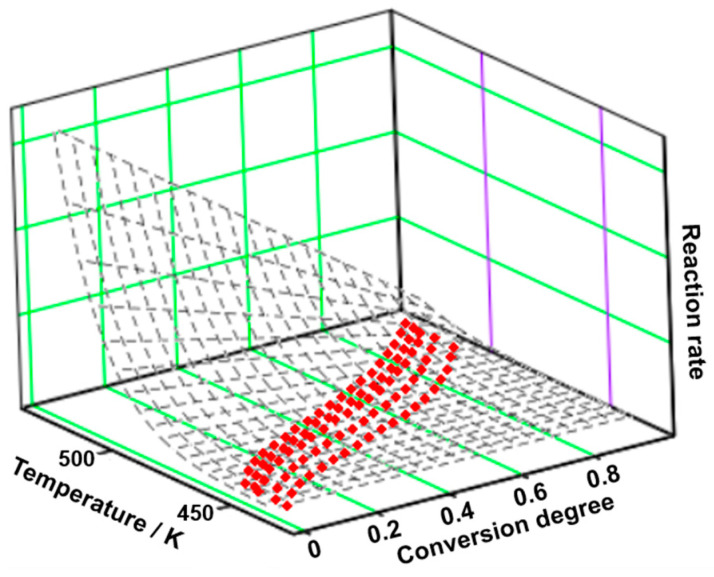
The 3D transformation surface for VLS from the NPK method.

Regarding the graphical representation (Figure 6) and the results of the NPK analysis of VLS (Table 7), the degradation process consists of two parallel processes, the obtained value of the activation energy being the result of using the two values of variance (λ).

The greatest contribution to the degradation process is presented by the first process, which displays a variance of λ = 91.4% and which is a chemical reaction with a reaction order of n = 1. For the second process (λ = 8.3%), even if its contribution to the global process is remarkably reduced, the value of the activation energy is similar, and it consists only of physical transformations (m = 1).

**Figure 7:**
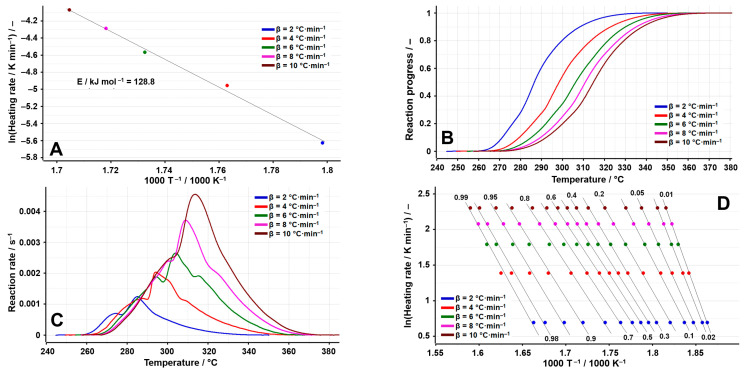

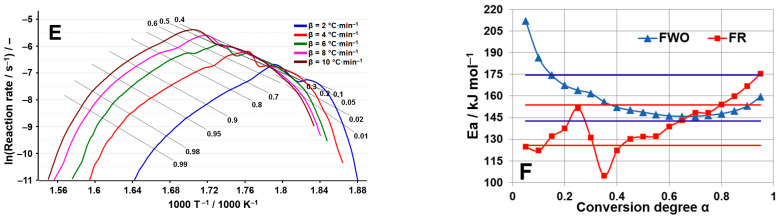
Results of the kinetic analysis performed on LOS: (**A**) kinetic method ASTM E698; (**B**) reaction progress vs. temperature; (**C**) reaction rate vs. temperature; (**D**) kinetic method FWO; (**E**) kinetic method Fr; (**F**) E_a_ vs. α according to the FWO and FR methods.

With reference to LOS, the analyzed process is in the range of 248–348 °C at a value of the heating rate of β = 2 °C min^−1^, with a value of the activation energy of 128.8 kJ mol^−1^ observed by using the ASTM E698 method, similar to the one obtained for VLS, being an indication of comparable stability between the two. The results of the kinetic investigation are presented in Figure 7A–F.

The isoconversional methods also indicate the complex degradation mechanism of the LOS: if for the FWO method a rapid decrease in E_a_ values is observed up to α = 0.25, after which the values remain within ±10% around the mean, for the FR method the variation is non-monotonic, confirming the complexity of the decomposition process, a hypothesis also sustained by the values presented in Table 5. Those results are expected, namely a complex decomposition mechanism, given its salt structure.

**Figure 8:**
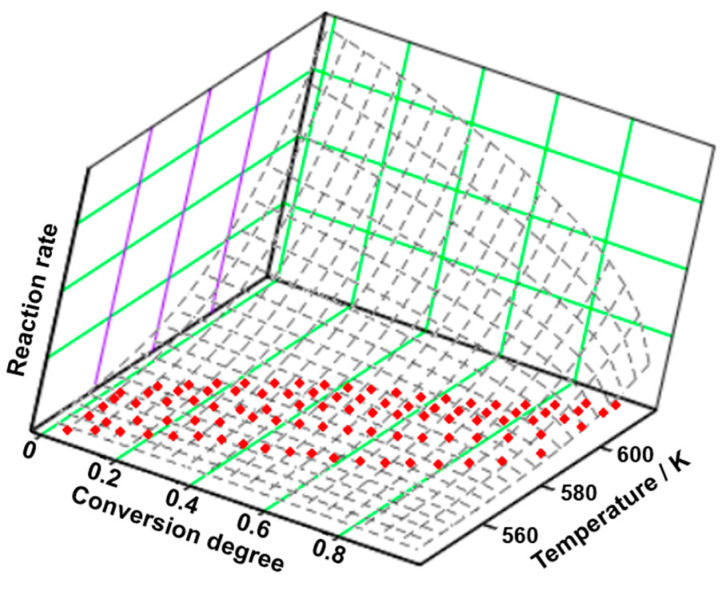
The 3D transformation surface for LOS from the NPK method.

In Figure 8 and Table 8, the results of the NPK method are presented. As can be observed, the main degradation process consists of two simultaneous processes, the first one being the main contributor to the process, with a variance of 89.4%. The value of the global activation energy is determined by considering the contribution of each stage to the main process, following the equation $\bar{E}=\lambda_{1}\cdot E_{1}+\lambda_{2}\cdot E_{2}$. The beginning of the degradation consists only of chemical degradations, given the reaction order of n = 2/5, which take place along with the chemical degradations (n = 3/2) and physical transformations (m = 1) of the second process.

**Figure 9:**
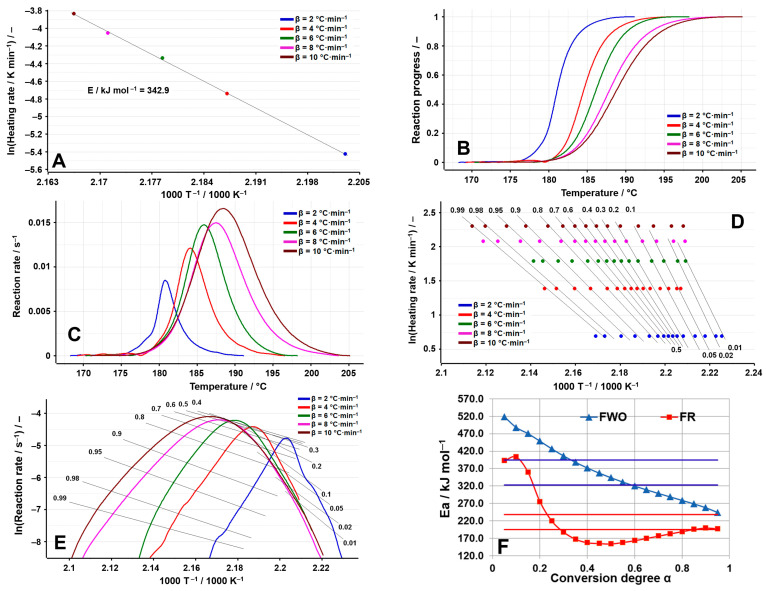
Results of the kinetic analysis performed on OLM dehydration: (**A**) kinetic method ASTM E698; (**B**) reaction progress vs. temperature; (**C**) reaction rate vs. temperature; (**D**) kinetic method FWO; (**E**) kinetic method Fr; (**F**) E_a_ vs. α according to the FWO and FR methods.

Regarding OLM, two distinct processes were analyzed from a kinetic point of view to obtain a better understanding of the degradation mechanism. The first analyzed process is represented by dehydration, since OLM is used as a hemihydrate salt, and the results are presented in Figure 9A–F.

The ASTM E698 method reveals an unusually high activation energy of 342.9 kJ mol^−1^, which is not typical for oxidative decomposition processes in pharmaceutical compounds. Two isoconversional methods, FR and FWO, indicate even higher values, as presented in Table 4 and Figure 9F. Specifically, the FWO method shows a progressive decrease in E_a_ values as the reaction proceeds, ranging from an extreme 518.8 kJ mol^−1^ at α = 5% to 244.2 kJ mol^−1^ at α = 95%. For this hemihydrate, the substantial and consistent variation in E_a_ can be attributed to the effects of mass and heat transfer. Examining the olmesartan medoxomil–water system at a macroscopic scale suggests that a single water molecule is associated with two active pharmaceutical molecules. In molar terms, 18 g of water is “bound” or “released” by 2 × 558.6 g of active ingredient, totaling 1117.2 g. This integration of water molecules within the OLM molecular network complicates the initiation of mass and heat transfer, thus necessitating higher energies until a steady-state diffusion of water molecules from the solid can be reached. During dehydration, local fluctuations in water vapor pressure and temperature may occur, potentially distorting kinetic analysis results and leading to misleading activation energy dependencies as conversion increases [42,43].

**Figure 10:**
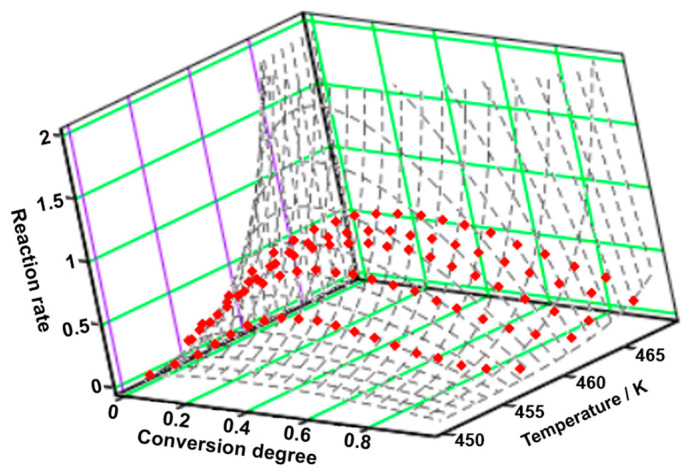
The 3D transformation surface for dehydration of OLM from the NPK method.

By applying the NPK method, the tridimensional graphic of the experimental points together with the interpolation of the reaction rate as a continuous surface was obtained (Figure 10). The dehydration of OLM consists of two concomitant steps, the first one with λ = 91% and the second one with λ = 8.4% (see Table 9). In the matter of these two processes, they are the result of both physical transformations (m = 1/2) and chemical degradations (n = 1).

**Figure 11:**
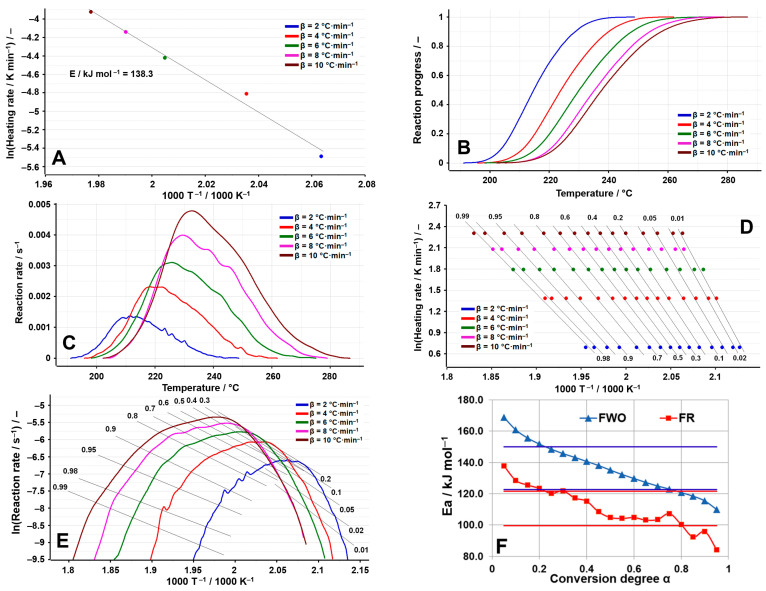
Results of the kinetic analysis performed on OLM degradation: (**A**) kinetic method ASTM E698; (**B**) reaction progress vs. temperature; (**C**) reaction rate vs. temperature; (**D**) kinetic method FWO; (**E**) kinetic method Fr; (**F**) E_a_ vs. α according to the FWO and FR methods.

By analyzing the decomposition process of OLM (Figure 11A–F), a pattern similar to that observed during dehydration is noted when examining the variation in activation energies through the FWO method. The decomposition of anhydrous OLM involves multiple competing processes, a hypothesis further supported by the graphical representation of the reaction rate curves vs. T^−1^ (Figure 11C). The variation seen in Figure 11F can be attributed to the diffusion of water vapor from the solid matrix of the active pharmaceutical ingredient. This diffusion leads to continuous changes in the reactivity of the reactant particles, Influenced by alterations In the crystalline structure, the emergence of defects at interstitial sites, their migration, and other related phenomena.

**Figure 12:**
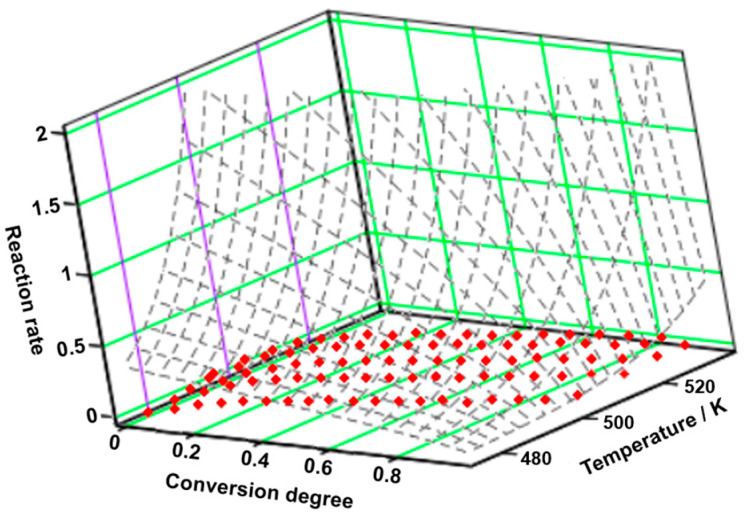
The 3D transformation surface for degradation of OLM from the NPK method.

By applying the NPK method, it was obtained the tridimensional graphic of the experimental points together with the interpolation of the reaction rate as a continuous surface (Figure 12). Concerning the degradation of OLM, it can be observed that the major contributor to the process is the first step, presenting a variance of 94.2% (see Table 10). The transformations during this step are only chemical (n = 4/5), while for the second step both are observed (physical, m = 2/3, and chemical, n = 1).

## 3. Materials and Methods

### 3.1. Samples

The active pharmaceutical ingredients used were commercially produced and used without any further purification in this study, presenting the following provenance and purity:

- TELM was obtained from Sigma-Aldrich (St. Louis, MO, USA), product code PHR1855, secondary pharmaceutical standard;
- VLS was obtained from Sigma-Aldrich (St. Louis, MO, USA), product code PHR1315, secondary pharmaceutical standard, certified reference material;
- OLM hemihydrate was obtained from Sigma-Aldrich (St. Louis, MO, USA), product code SML1391, presenting a purity of ≥98% (HPLC);
- LOS was obtained from Sigma-Aldrich (St. Louis, MO, USA), product code PHR1602, secondary pharmaceutical standard, certified reference material.

### 3.2. Thermal Investigations

The thermal investigations were performed on the Setline TGA (SETARAM, Caluire, France) instrument using an open alumina crucible in a dynamic air atmosphere (100 mL min^−1^), to obtain the TG and DTG data. Five different heating rates were selected, namely β = 2, 4, 6, 8, and 10 °C min^−1^ from ambient temperature up to 800 °C, for a sample weighing approximately 7 mg. Regarding the DSC curves, a NETZSCH DSC 204F1 Phoenix (NETZSCH, Selb, Germany) instrument and sealed aluminum crucibles were used, in an inert nitrogen medium with a flow rate of 20 mL min^−1^ and a heating rate of 10 °C min^−1^.

### 3.3. Kinetic Analysis

The kinetic study (using the ASTM E698, FR, and FWO methods) was performed on the main decomposition step using AKTS Thermokinetics software, Version 4.46 (AKTS AG TechnoArk, Siders, Switzerland). The mathematical background and importance of using isoconversional kinetic methods have been extensively reported in the literature [44,45,46,47,48]. Also, to obtain the values for the kinetic triplet, the modified non-parametric method (NPK) was used, through a protocol that was previously reported by our research group [37,38,39].

The abbreviations used are those recommended and accepted by the ICTAC committee [49,50]: α—degree of conversion, t—time, β—linear heating rate (°C min^−1^), A—pre- exponential factor, according to the Arrhenius kinetic model (min^−1^), f(α)—differential conversion function, g(α)—integral conversion function, E_a_—activation energy (kJ mol^−1^), R—universal gas constant (J mol^−1^ K^−1^), T—absolute temperature (K), m_o_—mass of the sample at the beginning of decomposition, m_f_—mass of the sample at the end of decomposition, and m_T_—mass of the sample at a certain temperature T.

## 4. Conclusions

From the results of this study, it can be concluded that all the selected sartans undergo a complex decomposition process, consisting of complex reaction sequences (processes involving parallel and successive reactions, processes with reversible steps, and processes with a transition to the diffusion regime); the curves E_a_ vs. α show minimal and maximal values and ranges where E_a_ is independent of the conversion. It is also worth mentioning that the change in the decomposition mechanism with the increase in the heating rate occurs for all investigated sartans, the variation in the individual-point E_a_ values being in wide intervals outside the limit interval of 10% variation around the average, and therefore, the average value tabulated for the activation energy only has the mathematical meaning of an average value and does not represent the average energy of a unitary, individualized process.

## Figures and Tables

**Table 1:** Physicochemical properties of olmesartan medoxomil, telmisartan, losartan potassium, and valsartan.

	Olmesartan Medoxomil	Telmisartan	Valsartan	Losartan Potassium
Chemical formula	C_29_H_30_N_6_O_6_	C_33_H_30_N_4_O_2_	C_24_H_29_N_5_O_3_	C_22_H_22_ClKN_6_O
Molar mass/g mol^−1^	558.6	514.6	435.5	461.0
Aggregation state	Solid	Solid	Solid	Solid
Melting point/°C	128–148 153–165 177–180 [12]	261–263	116–117	268–271 [13]
Solubility * at 25 °C /mg L^−1^	Water: p.i. (<2.32·10^−7^) [14]	Water: p.i. (2.8·10^−6^); Strong acids: s.s. (except for HCl: p.i.) Strong bases: s. [10]	Water: s.s.; EtOH, DMSO, DMF: s. [15]	Water: s. MeOH, EtOH, 1-PrOH, 2-PrOH DMSO, DMF: s. [13,16]

* p.i. = practically insoluble; s.s. = slightly soluble; s = soluble.

**Table 2:** The interpretation of the thermoanalytical curves.

Sample	Process	T_onset_ (°C)	T_offset_ (°C)	T_onset DTG_ (°C)	T_max DTG_ (°C)	T_onset DSC_ (°C)	T_peak DSC_ (°C)	ΔH_fus_ (J g^−1^)	Δm (%)
TELM	I	274	419	274	392; 424	256	271	103.5	38.8
	II	450	668	466	569	-	-	-	59.7
OLM	I	171	202	171	189	172	185	122.8	3.4
	II	202	287	202	232	-	-	-	21.7
	III	287	353	287	297; 327	-	-	-	7.4
	IV	353	504	353	367; 387; 441	-	-	-	22.9
	V	504	690	515	575	-	-	-	44.7
LOS	I	258	380	258	314	232	240	9.3	25.4
						263	275	99.4	
	II	418	538	420	510	-	-	-	19.3
	III	538	652	540	594	-	-	-	34.6
VLS	I	45	108	45	80	92	103	26.4	0.5
	II	148	277	148	212	-	-	-	13.3
	III	284	486	287	393	-	-	-	52.9
	IV	486	662	493	575	-	-	-	32.4

**Table 3:** The temperature ranges for the first decomposition processes for each sartan for the kinetic investigations.

β (°C min^−1^)	The Temperature Range for the Selected Process (°C)				
	LOS	VLS	TELM	OLM Dehydration	OLM Decomposition
2	248–348	136–251	266–359	168–191	191–249
4	255–363	138–260	268–387	169–196	196–262
6	256–371	141–268	272–400	170–198	198–275
8	258–375	144–272	274–406	171–202	202–279
10	258–380	148–277	274–419	171–202	202–287

**Table 4:** The obtained values for the activation energy using the FWO and FR isoconversional methods [40].

	E_a_ (kJ mol^−1^) vs. α for									
	TELM		VLS		OLM				LOS	
					Dehydration		Decomposition			
α	FWO	FR	FWO	FR	FWO	FR	FWO	FR	FWO	FR
0.05	107.8	92.4	134.1	139.8	518.8	393.1	168.8	137.9	212.0	124.8
0.10	103.3	81.5	136.8	137.1	488.1	403.6	160.8	128.6	186.6	122.1
0.15	99.5	76.5	137.1	136.7	470.4	359.5	155.6	125.7	174.3	132.0
0.20	96.8	76.3	137.5	138.0	448.9	275.6	151.7	123.5	167.4	137.5
0.25	95.1	78.3	137.7	136.2	426.7	220.1	148.5	120.3	163.9	151.6
0.30	93.9	78.6	137.3	133.5	406.5	188.3	145.6	121.9	161.8	131.2
0.35	93.1	79.7	136.4	130.4	388.3	168.0	143.2	117.3	155.9	104.8
0.40	92.6	81.6	135.3	128.6	371.9	157.9	140.6	115.3	152.1	122.1
0.45	92.2	82.4	134.2	127.2	357.2	155.5	138.0	108.5	150.1	130.2
0.50	92.0	83.8	133.2	127.8	343.8	154.4	135.1	104.9	148.6	131.8
0.55	91.9	86.0	132.5	129.0	331.4	158.5	132.3	104.2	147.2	132.2
0.60	91.9	87.6	132.2	130.7	319.9	163.7	129.7	104.8	146.1	138.7
0.65	92.0	90.0	132.6	135.2	309.1	170.2	127.2	103.2	145.8	143.3
0.70	92.2	91.5	134.1	141.1	298.8	176.7	124.9	103.6	145.8	148.3
0.75	92.6	93.5	136.8	148.8	288.8	183.2	123.0	107.4	146.5	148.4
0.80	93.0	96.0	141.8	158.7	278.9	189.6	120.7	100.3	147.6	154.1
0.85	93.6	98.4	148.2	166.1	268.8	195.8	118.4	92.4	149.6	159.8
0.90	94.4	101.1	155.5	173.2	257.9	199.9	115.4	95.9	153.0	166.9
0.95	95.6	106.6	163.0	177.4	244.2	197.8	109.9	84.2	159.4	175.3
$\bar{E}$_a_ (kJ mol^−1^)	94.9 ± 1.0	87.5 ± 2.0	138.8 ± 1.9	141.9 ± 3.6	358.9 ± 18.9	216.4 ± 18.5	136.3 ± 3.7	110.5 ± 3.1	158.6 ± 3.9	139.7 ± 3.9

**Table 5:** The estimation of the complexity of the analyzed processes based on the values of the activation energies.

	Kinetic Method	E_max_	E_min_	${\bar{E}}_{a}$	E_max_−E_min_	$20\%\cdot {\bar{E}}_{a}$
TELM	FWO	107.8	91.9	94.9	15.9	19.0
	FR	106.6	76.3	87.5	30.3	17.5
VLS	FWO	163	132.2	138.8	30.8	27.8
	FR	177.4	127.2	141.9	50.2	28.4
OLM Dehydration	FWO	518.8	244.2	358.9	274.6	71.8
	FR	403.6	154.4	216.4	249.2	43.3
OLM Degradation	FWO	168.8	109.9	136.3	58.9	27.3
	FR	137.9	84.2	110.5	53.7	22.1
LOS	FWO	212	145.8	158.6	66.2	31.7
	FR	175.3	104.8	139.7	70.5	27.9

**Table 6:** Results of the NPK method for analysis of TELM and comparison with isoconversional methods.

Sample	Process	λ/%	E/kJ mol^−1^	A/min^−1^	n	m	R^2^	f(α)	$\bar{E}$_a_ (kJ mol^−1^)		
									NPK	FWO	FR
TELM	1	87.5	85.7 ± 1.2	3.4·10^7^ ± 1.1·10^4^	1/2	1	0.950	(1–x)^1/2^ · x^1^	82.6 ± 1.2	87.5 ± 2.0	94.9 ± 1.0
	2	8.6	89.5 ± 0.2	1.3·10^7^ ± 6.6	1	3/2	0.955	(1–x)^1^ · x^3/2^			

**Table 7:** Results of the NPK method for analysis of VLS and comparison with isoconversional methods.

Sample	Process	λ/%	E/kJ mol^−1^	A/min^−1^	n	m	R^2^	f(α)	$\bar{E}$_a_ (kJ mol^−1^)		
									NPK	FWO	FR
VLS	1	91.4	136.3 ± 2.5	3.0·10^15^ ± 6.8·10^10^	1	0	0.993	(1–x)^1^	136.4 ± 2.9	138.8 ± 1.9	141.9 ± 3.6
	2	8.3	142.4 ± 0.3	1.9·10^16^ ± 8.7·10^6^	0	1	0.986	x^1^			

**Table 8:** Results of the NPK method for analysis of LOS and comparison with isoconversional methods.

Sample	Process	λ/%	E/kJ mol^−1^	A/min^−1^	n	m	R^2^	f(α)	$\bar{E}$_a_ (kJ mol^−1^)		
									NPK	FWO	FR
LOS	1	89.4	158.6 ± 2.2	6.1·10^14^ ± 8.8·10^7^	2/5	0	0.983	(1–x)^2/5^	151.3 ± 2.4	139.7 ± 3.9	158.6 ± 4.1
	2	7.0	138.5 ± 0.3	2.6·10^11^ ± 1.4·10^11^	3/2	1	0.995	(1–x)^3/2^ · x^1^			

**Table 9:** Results of the NPK method for analysis of dehydration of OLM and comparison with isoconversional methods.

Sample	Process	λ/%	E/kJ mol^−1^	A/min^−1^	n	m	R^2^	f(α)	$\bar{E}$_a_ (kJ mol^−1^)		
									NPK	FWO	FR
Dehydration of OLM	1	91.0	461.8 ± 2.7	1.0·10^53^ ± 2.8·10^12^	1	1/2	0.987	(1–x)^1^ · x^1/2^	441.4 ± 2.9	358.9 ± 18.9	216.4 ± 18.5
	2	8.4	252.4 ± 0.2	2.0·10^28^ ± 1.5·10^8^	1	1/2	0.943	(1–x)^1^ · x^1/2^			

**Table 10:** Results of the NPK method for analysis of degradation of OLM and comparison with isoconversional methods.

Sample	Process	λ/%	E/kJ mol^−1^	A/min^−1^	n	m	R^2^	f(α)	$\bar{E}$_a_ (kJ mol^−1^)		
									NPK	FWO	FR
Degradation of OLM	1	94.2	136.5 ± 1.6	4.0·10^14^ ± 2.1·10^6^	4/5	0	0.997	(1–x)^4/5^	133.9 ± 13.6	110.5 ± 3.1	136.3 ± 3.7
	2	3.8	141.4 ± 0.1	5.7·10^13^ ± 1.2·10^4^	1	2/3	0.991	(1–x)^1^ · x^2/3^			

## Data Availability

Raw data are available upon request from the corresponding author of this work.
